# Dual Inhibitors of Amyloid-β and Tau Aggregation with Amyloid-β Disaggregating Properties: Extended *In Cellulo*, *In Silico*, and Kinetic Studies of Multifunctional Anti-Alzheimer’s Agents

**DOI:** 10.1021/acschemneuro.1c00235

**Published:** 2021-05-21

**Authors:** Anna Pasieka, Dawid Panek, Natalia Szałaj, Alba Espargaró, Anna Więckowska, Barbara Malawska, Raimon Sabaté, Marek Bajda

**Affiliations:** † Department of Physicochemical Drug Analysis, Faculty of Pharmacy, Jagiellonian University Medical College, Medyczna 9, 30-688 Krakow, Poland; ‡ Department of Pharmacy and Pharmaceutical Technology and Physical-Chemistry, School of Pharmacy and Food Sciences, University of Barcelona, Av Joan XXIII 27-31, 08028 Barcelona, Spain; § Institute of Nanoscience and Nanotechnology (IN2UB), Av Joan XXIII, S/N, 08028 Barcelona, Spain

**Keywords:** β-Amyloid, tau protein, Alzheimer’s disease, aggregation inhibitors, molecular modeling

## Abstract

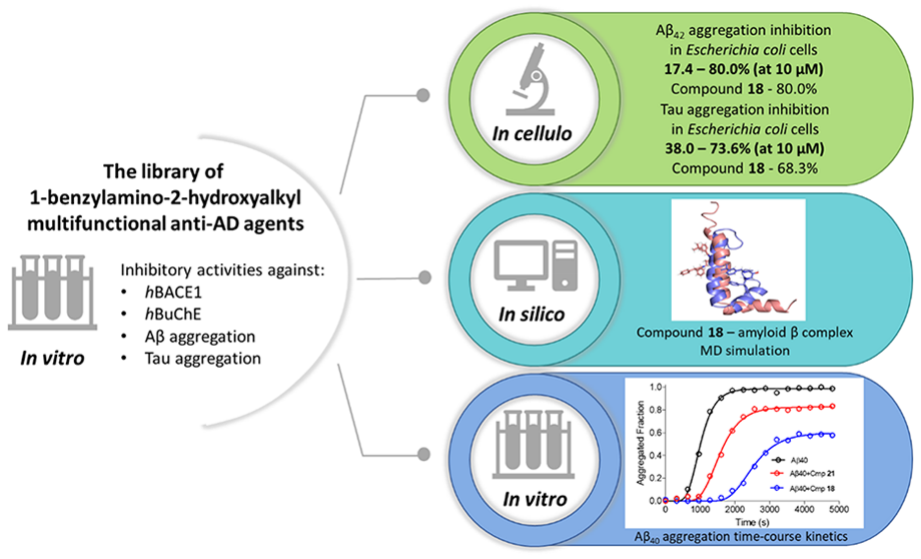

In Alzheimer’s disease, neurons slowly degenerate due to the accumulation of misfolded amyloid β and tau proteins. In our research, we performed extended studies directed at amyloid β and tau aggregation inhibition using *in cellulo* (*Escherichia coli* model of protein aggregation), *in silico*, and *in vitro* kinetic studies. We tested our library of 1-benzylamino-2-hydroxyalkyl multifunctional anti-Alzheimer’s agents and identified very potent dual aggregation inhibitors. Among the tested derivatives, we selected compound **18**, which exhibited a unique profile of biological activity. This compound was the most potent and balanced dual aggregation inhibitor (Aβ_42_ inhibition (inh.) 80.0%, tau inh. 68.3% in 10 μM), with previously reported *in vitro* inhibitory activity against *h*BuChE, *h*BACE1, and Aβ (*h*BuChE IC_50_ = 5.74 μM; *h*BACE1 IC_50_ = 41.6 μM; Aβ aggregation (aggr.) inh. IC_50_ = 3.09 μM). In docking studies for both proteins, we tried to explain the different structural requirements for the inhibition of Aβ vs tau. Moreover, docking and kinetic studies showed that compound **18** could inhibit the amyloid aggregation process at several steps and also displayed disaggregating properties. These results may help to design the next generations of dual or selective aggregation inhibitors.

## 1 Introduction

The accumulation of misfolded proteins causes common neurodegenerative diseases such as Alzheimer’s disease (AD), Parkinson’s disease, and Huntington’s disease, as well as type II diabetes.^1^ In AD, neurons slowly degenerate and lose their functions due to the accumulation of amyloid β (Aβ) plaques and tau tangles (NFTs) in the brain.^2^ The lack of effective treatment and aging of societies have caused a continuous increase in the number of patients and fatalities caused by AD.^3^ The current treatment of AD is limited to four drugs that mainly treat the symptoms of the disease and do not show disease-modifying effects. These drugs modulate the cholinergic (donepezil, rivastigmine, galantamine) and glutamatergic (memantine) neurotransmission systems and only slightly improve memory functions.^4^ Thus, in modern drug discovery programs, efforts are focused on disease-modifying pharmacological targets. In 2020, 97 out of 121 clinical agents were in disease modification trials.^5^

Approximately 20% of compounds in the currently ongoing clinical trials for AD target Aβ and tau proteins.^5^ In Aβ/tau-oriented projects, the focus is placed on reducing Aβ/tau formation and aggregation or inducing the removal of already formed deposits of proteins.^6^ Such effects can be obtained by using protein aggregation inhibitors, antibodies, and enzyme inhibitors. Several dual Aβ and tau aggregation inhibitors have recently been published in the literature.^7−10^ Okuda et al. reported a curcumin derivative, PE859, which acts as a dual aggregation inhibitor that ameliorates cognitive dysfunction in senescence-accelerated mouse-prone 8,^8^ an animal model that displays a phenotype of accelerated aging. Additionally, recent positive outcomes from the anti-Aβ monoclonal antibody aducanumab, which is currently undergoing review by the Food and Drug Administration (FDA), give hope that antiamyloid therapies may be a promising alternative to the currently available AD treatment.^11^

Lowered Aβ levels can also be achieved by inhibition of β-secretase (BACE1) and γ-secretases that are responsible for the so-called amyloidogenic pathway of amyloid precursor protein (APP) breakdown. BACE1 inhibitors entered clinical trials and were tested in patients at early and mild-to-moderate stages of AD with fully developed Aβ pathology.^12,13^ Elenbecestat (E-2609, Eisai),^14^ verubecestat (MK-8931, Merck),^15^ and atabecestat (JNJ-911, Janssen)^16^ reached phase III clinical trials but failed due to lack of clinical efficacy or observed toxicity.^12,17,18^

Although much effort has been put toward anti-AD drug development, many clinical trials for AD therapy with single-target drugs have recently failed. In complex diseases, where single-target drugs do not achieve the desired results, treatment combinations or multitarget drugs often result in higher effectiveness.^19,20^ Additionally, multitarget drugs offer a simpler dosage regimen and better patient compliance, which are of great importance in long-term treatment.^21^ The major concern with this approach is the choice of targets that we want to combine. This selection should be made carefully to obtain balanced activity against the targets of interest.^22^

Recently, we reported a series of multitarget-directed anti-AD agents with the potential to alleviate symptoms and treat causes of the disease.^23^ These 1-benzylamino-2-hydroxyalkyl derivatives exhibited a balanced profile of inhibitory activities against BACE1, butyrylcholinesterase (BuChE), and Aβ aggregation. Among them, seven compounds were found to be potent Aβ aggregation inhibitors *in vitro*, with a percent inhibition of more than 50% at a screening concentration of 10 μM. The most potent inhibitor had an IC_50_ value of 3.09 μM. We also tested four compounds against truncated and full-length forms of tau protein in a pilot *in vitro* study. These compounds inhibited tau aggregation in the range 45–70% at 10 μM. The promising preliminary results of Aβ and tau protein inhibition *in vitro* encouraged us to continue our investigation of the antiaggregating properties of this library of compounds and to find the structure–activity relationship within this group.

Herein, we present our extended study on the activity of 1-benzylamino-2-hydroxyalkyl multifunctional anti-AD agents using an *in cellulo* thioflavin S (ThS) assay in recombinant *Escherichia coli* cells overexpressing Aβ_42_ peptide and tau proteins.^24^ We also performed molecular modeling studies to investigate possible interactions of the tested compounds with Aβ and tau proteins and for selected compounds kinetic aggregation studies and dissagregation studies *in vitro* using Aβ_40_.

## 2 Results and Discussion

### 2.1 Biological Evaluation

#### 2.1.1 Compound Library

**Figure 1:**
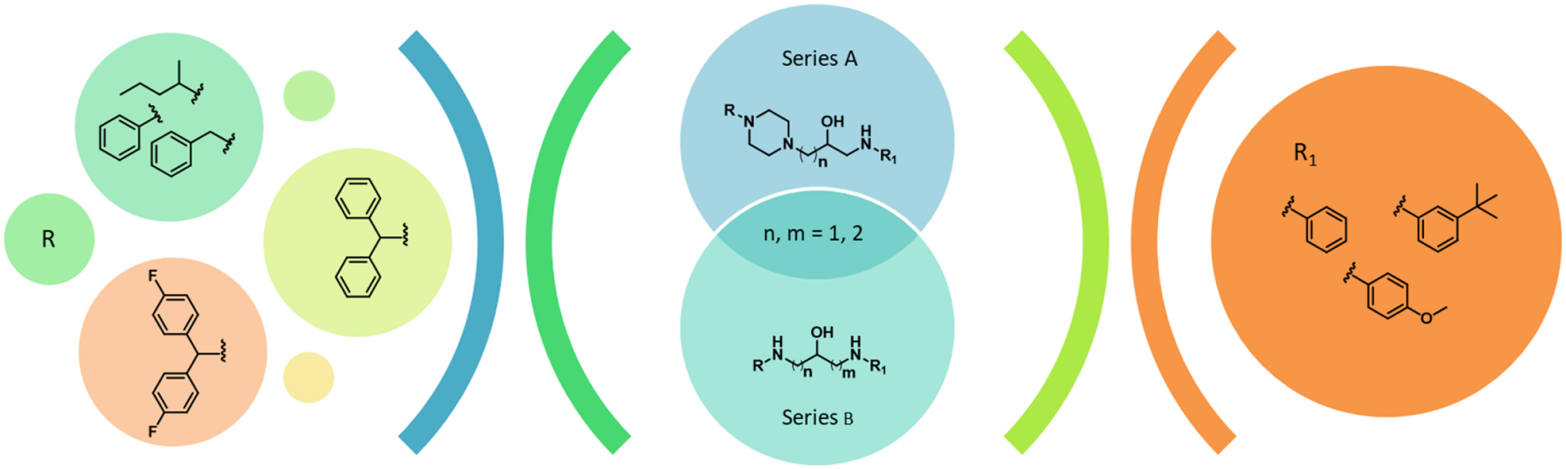
Test library of multifunctional 1-benzylamino-2-hydroxyalkyl derivatives.

The library of 24 previously synthesized compounds,^23^ which was selected for broadened *in cellulo* aggregation studies, contains two series (series A and B) of benzylamine-hydroxyalkylamine derivatives (Figure 1). In series A, the hydroxyalkylamine nitrogen atom was incorporated into a piperazine ring. The piperazine ring in series A and the hydroxyalkylamine nitrogen atom in series B were substituted by diphenylmethyl, 2,2-diphenylethyl, 3,3-diphenylpropyl, *bis*(4-fluorophenyl)-methyl, phenyl, benzyl, or pentan-2-yl moieties, which were selected by virtual screening of the building blocks available from Sigma-Aldrich against BACE1, acetylcholinesterase (AChE), and BuChE. Notably, the majority of the compounds meet the criteria of druglikness.

#### 2.1.2 Inhibition of Aβ_42_ and Tau Aggregation

We tested 24 compounds from our library of multifunctional ligands using a fluorescence ThS assay *in cellulo*.^24^ In this assay, the changes in the fluorescence intensity level of ThS, which depend on the presence of β-sheet-rich structures such as β-amyloid and tau aggregates, were monitored. In contrast to the *in vitro* assay, where synthetic proteins were used, we used recombinant *E. coli* bacteria overproducing Aβ_42_ or full-length tau. Insoluble aggregates of Aβ and tau, called inclusion bodies (IBs), are found inside bacterial cells when the bacteria are forced to produce heterologous proteins.^25,26^ Comparing induced and noninduced cells with or without potential inhibitors allows us to evaluate the influence of the tested compounds on tau and Aβ aggregation. The method is simple, fast, and inexpensive, but it requires the compounds to cross the bacterial membranes, which creates a risk of not detecting potential inhibitors.^27^ Despite this inconvenience, conducting experiments using bacterial cells has a great advantage: The conditions of the aggregation process are more comparable and closer to those of mammals than those of *in vitro* assays.^28^ This method has already been validated and used in an efficient evaluation of antiaggregating properties for a series of compounds in several drug discovery projects.^29−32^ As a reference compound, we used DP-128 whose Aβ and tau antiaggregating properties were revealed *in cellulo* earlier.^27,33^ Some of the investigated compounds were previously tested in PAMPA assay (parallel artificial membrane permeability assay).^23^ They were representatives of both series. As all of them showed a high probability for penetration of the membrane, we can assume that all derivatives from this library have the same ability and can be tested in *in cellulo* studies. It is worth mentioning that, in the case of this library, the risk of not detecting potential inhibitors due to the lack of permeability is really low.

The results of our studies are presented in Table 1. At the 10 μM screening concentration, the compounds from our library displayed moderate to potent dual antiaggregating properties with predominance toward the inhibition of tau aggregation, with percentages of inhibition in the ranges 17.4–80.0% (Aβ_42_ aggregation) and 38.0–73.6% (tau aggregation). Nineteen out of 24 compounds inhibited tau aggregation by more than 50%, whereas only 8 compounds inhibited Aβ, underlying the different structural requirements for strong inhibition of both proteins.

**Table 1:**
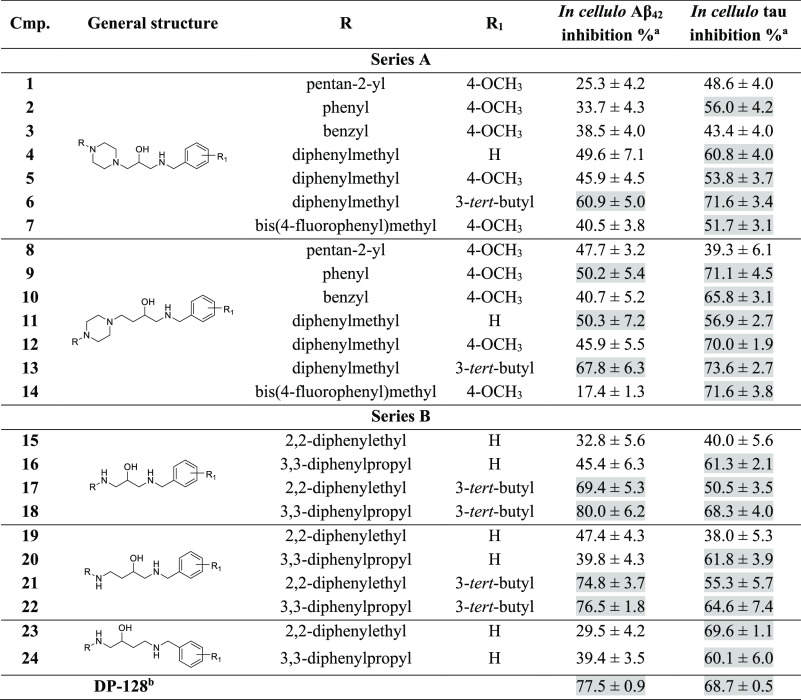
Inhibitory Activity against Aβ_42_ Peptide and Tau Protein Aggregation in *E. coli* Cells for Compounds **1–14** (Series A) and **15–24** (Series B)

a The percent inhibition at 10 μM (mean of three experiments ± SEM). Compounds with percent of inhibition above 50% are highlighted in gray.

b Reference (33).

Analyzing the structure–activity relationship in series A, we observed similar trends for both activities. First, we noticed that larger and bulkier moieties such as the diphenylmethyl group in the R fragment were more beneficial for aggregation inhibition than pentanyl, phenyl, or benzyl fragments (**5** vs **1**, **2**, **3**). Moreover, the compounds with a longer alkyl chain between the hydroxyl group and nitrogen atom from piperazine were found to be more active (**9** vs **2**, **10** vs **3**). Considering the influence of the benzylamine substitution, we pointed out that hybrids with a *tert*-butyl substituent at the *meta* position were the most potent inhibitors (**6** vs **4**, **5** and **13** vs **11**).

The comparison of results for both series showed that the piperazine derivatives possessed stronger tau antiaggregating properties than those of homologues with an open-ring fragment (**4** vs **15**, **6** vs **17**, **11** vs **19**, **13** vs **21**). In the case of Aβ aggregation, this relationship was noticed in only two pairs (**4** vs **15** and **11** vs **19**).

The structure–activity studies in series B provided few relevant observations. First, when analyzing the impact of R substituents, it was clear that the replacement of the diphenylethyl fragment with a diphenylpropyl group improved antiaggregating properties for both proteins (**15** vs **16**, **17** vs **18**). This result indicates that this substitution may also improve the activity of compounds in series A after further optimization.

Next, we analyzed the influence of the length of the alkyl chain, and we did not observe significant differences in the activity. There was only one exception: compound **23**. Here, we observed that the location of the nitrogen atom of the benzylamine fragment played an important role in tau aggregation inhibition. The longer distance between a nitrogen atom of benzylamine and a hydroxyl group increased the inhibitory activity from 38.0% (**19**) to 69.6% (**23**). Finally, we also confirmed that, in this series, the substitution of the *tert*-butyl moiety at position 3 of benzylamine boosted the inhibition of aggregation of both proteins, with **18** representing the most potent inhibitor (Aβ_42_ inh. 80.0%, tau inh. 68.3%).

### 2.2 *In Silico* Studies

#### 2.2.1 Influence on Amyloid-β Aggregation

**Figure 2:**
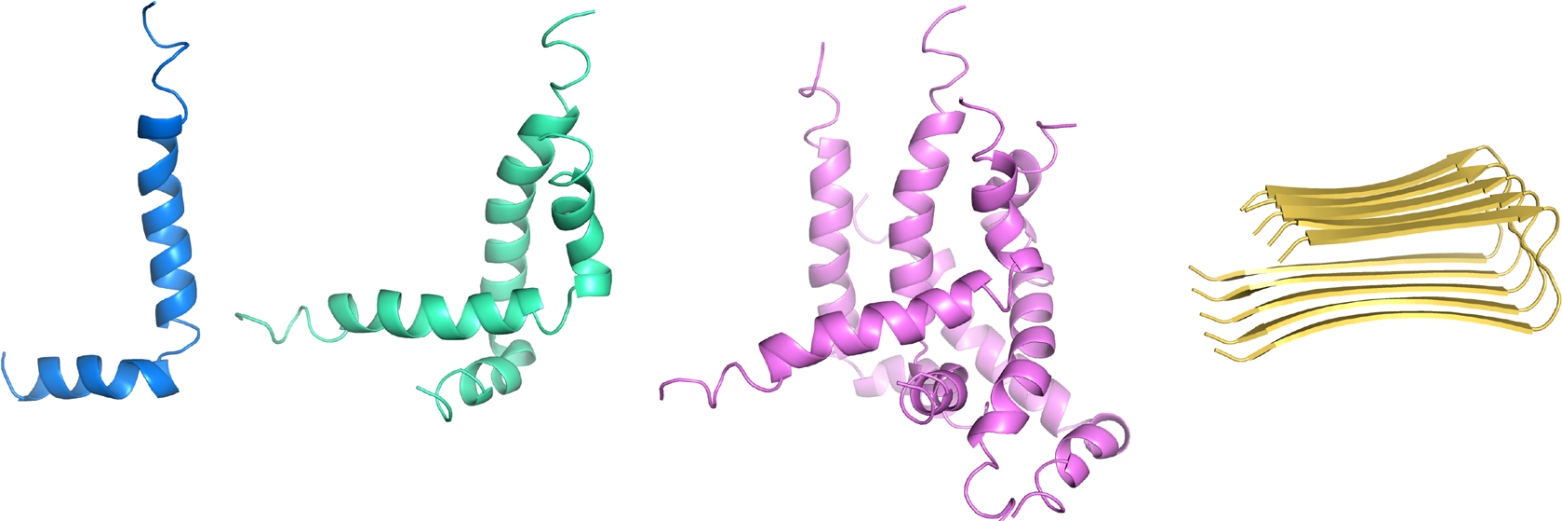
Different structures of amyloid-β used for the analysis of ligand binding: blue = helical monomer, green = dimer, violet = pentamer, gold = β-sheet pentamer.

The studied compounds revealed various levels of activity on amyloid aggregation. Therefore, we applied molecular modeling to investigate how they interact with amyloid-β and to determine the reasons for the diverse inhibitory potency. The analysis concerned different forms of amyloid-β with respect to the secondary structure and number of chains. We took into account the helical monomer, dimer, and pentamer of Aβ_1–42_ as well as the β-sheet pentamer Aβ_17–42_ (Figure 2).

The choice of these forms was justified by the fact that the process of aggregation starts from the release of helical amyloid fragments from the cell membrane upon cleavage of amyloid precursor protein by β- and γ-secretase. Then, these α-helical fragments convert into oligomers and fibrils with a β-structure; i.e., normally soluble peptides are converted to insoluble, β-rich amyloid deposits.^34^ Moreover, it has been reported that fibrillogenesis involves an oligomeric α-helical intermediate.^35,36^ The α-helical monomeric structure and β-sheet pentamer were taken directly from the Protein Data Bank (both structures determined by NMR spectroscopy; PDB codes 1IYT and 2BEG, respectively),^37,38^ while the oligomeric α-helical structures were obtained by protein–protein docking (Figure 2), and upon further preparation, all forms were used for ligand docking. The helical monomer and oligomers contained the whole 42-amino acid amyloid sequence (Aβ_1–42_), i.e., DAEFRHDSGY EVHHQKLVFF AEDVGSNKGA IIGLMVGGVV IA, and each chain presented an α-helix-kink-α-helix motif. In the case of fibrils, the N-terminal residues 1–17 were unorganized, while residues 18–42 created a β-strand (18–26)-turn-β-strand (31–42) motif. The whole structure was stabilized by intermolecular backbone hydrogen bonds in β-sheets and intermolecular interactions between side chains: a salt bridge with D23-K28, π–π stacking interactions with F19–F19 or F20–F20, and intermolecular knob-hole contacts in pairs F19-G38 and A21-V36. While building the α-helical dimeric structure, it was found that the polypeptide chains are organized generally in a parallel way, and this dimer is stabilized by side-chain hydrophobic interactions, especially between F20, V24, I31, and L34 from chain A and I31, F20, F19, and E22 from chain B, respectively. In the case of α-helical pentamers, we observed both parallel and antiparallel orientations of chains.

**Figure 3:**
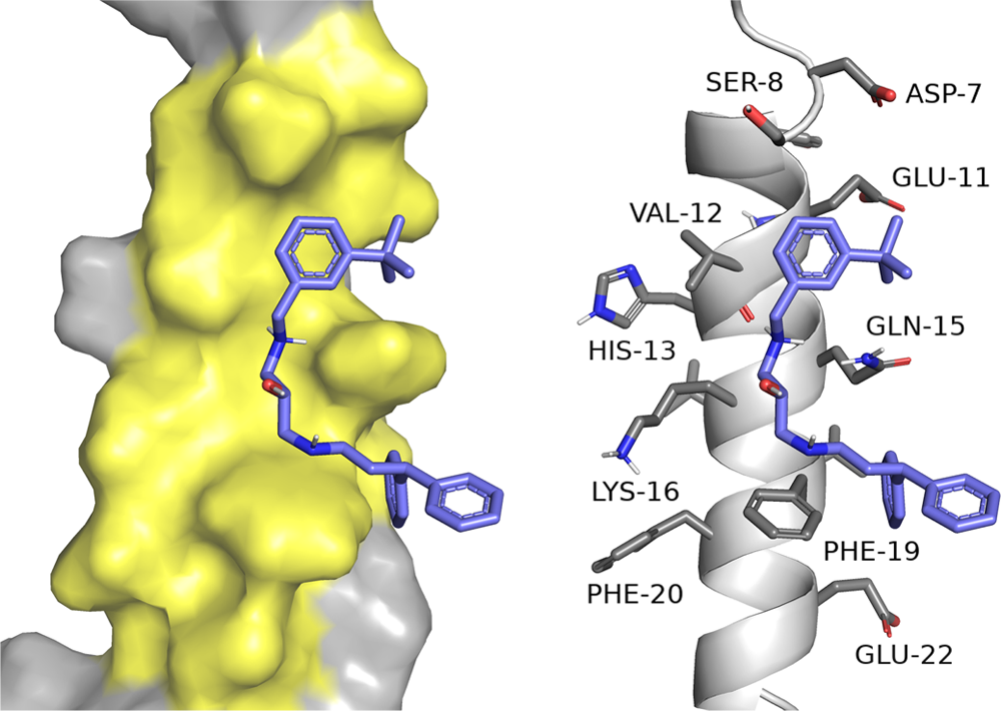
Binding mode of the *S*-isomer for the most active compound **18**.

Docking of the tested compounds to all mentioned amyloid forms was performed. As the ligands possess a stereogenic center and the biological assays were performed for racemic mixtures, all stereoisomers were taken into account during the analysis. While docking to the helical monomer Aβ_1–42_, the compounds were arranged along the α-helix, stretched between D7 and V24 and interacting with amino acid side chains (Figure 3). The hydrophobic interactions with V12 and F19 were the most important and could prevent peptide–peptide hydrophobic interactions relevant for aggregation.^39^ The GoldScore values were generally consistent with the activity, and those for the active compounds (e.g., **18**, **21**, **22**) were higher than those for the inactive/low-activity compounds (e.g., **14**). Moreover, the *S*-stereoisomers received a higher docking score, which may suggest that these isomers are more active against amyloid-β.

**Figure 4:**
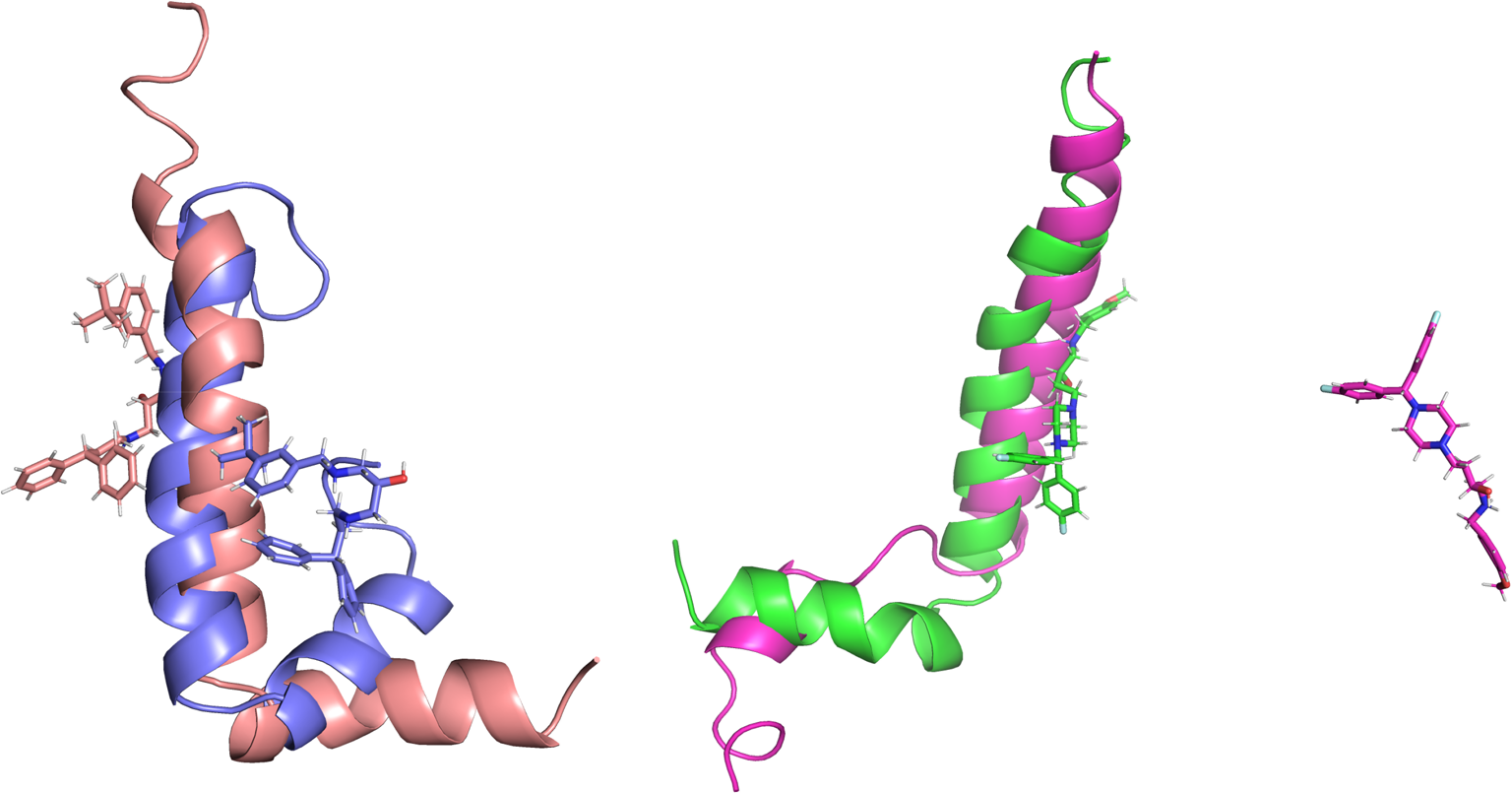
Comparison of the active compound (*S*)-**18** (left panel) and inactive (*S*)-**14** (right panel) over the course of MD simulation. Structures are coded by colors as follows: salmon = structure of the Aβ_1–42_ compound **18** complex at the beginning of MD simulation; violet = structure of the Aβ_1–42_ compound **18** complex upon 10 ns MD simulation; green = structure of the Aβ_1–42_ compound **14** complex at the beginning of MD simulation; pink = structure of the separated Aβ_1–42_ and compound **14** upon 10 ns MD simulation.

Furthermore, molecular dynamics simulations revealed a difference in the behavior of active (**18**) and inactive (**14**) compounds. Even though the ligand-amyloid-β complex underwent reorganization in the case of the potent compound **18**, it was quite stable, suggesting that this compound could stabilize the α-helical structure of amyloid; by contrast, the complex of the inactive derivative **14** with amyloid dissociated, separating amyloid and the compound from each other (Figure 4).

**Figure 5:**
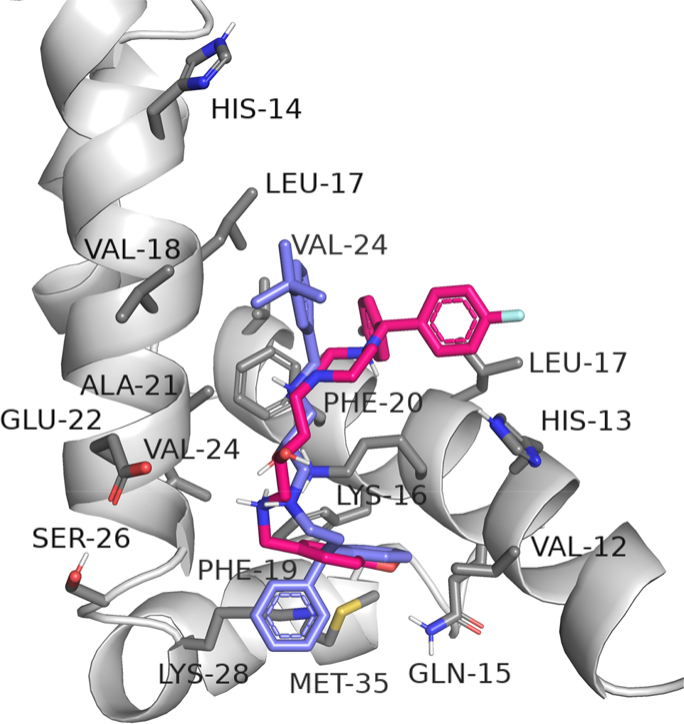
Binding mode of compounds (*S*)-**18** (violet) and (*S*)-**14** (pink) within the helical dimer.

During docking to the helical dimer, it was observed that both the active and inactive derivatives occupied the same area (Figure 5). However, the potent compound **18** was located in the opposite direction from that of inactive **14**. Except for hydrophobic interactions with L17, V18, A21, and V24 from chain A and V12, F16, and V24 from chain B, inhibitor **18** was able to create cation−π interactions via the benzhydryl moiety with K28 (chain A) and via the benzylamine group with F20 (chain B) as well as a hydrogen bond via the hydroxyl group with K16. It appears that the *S*-isomer might be more potent, because the *R*-isomer did not create the mentioned H-bond.

**Figure 6:**
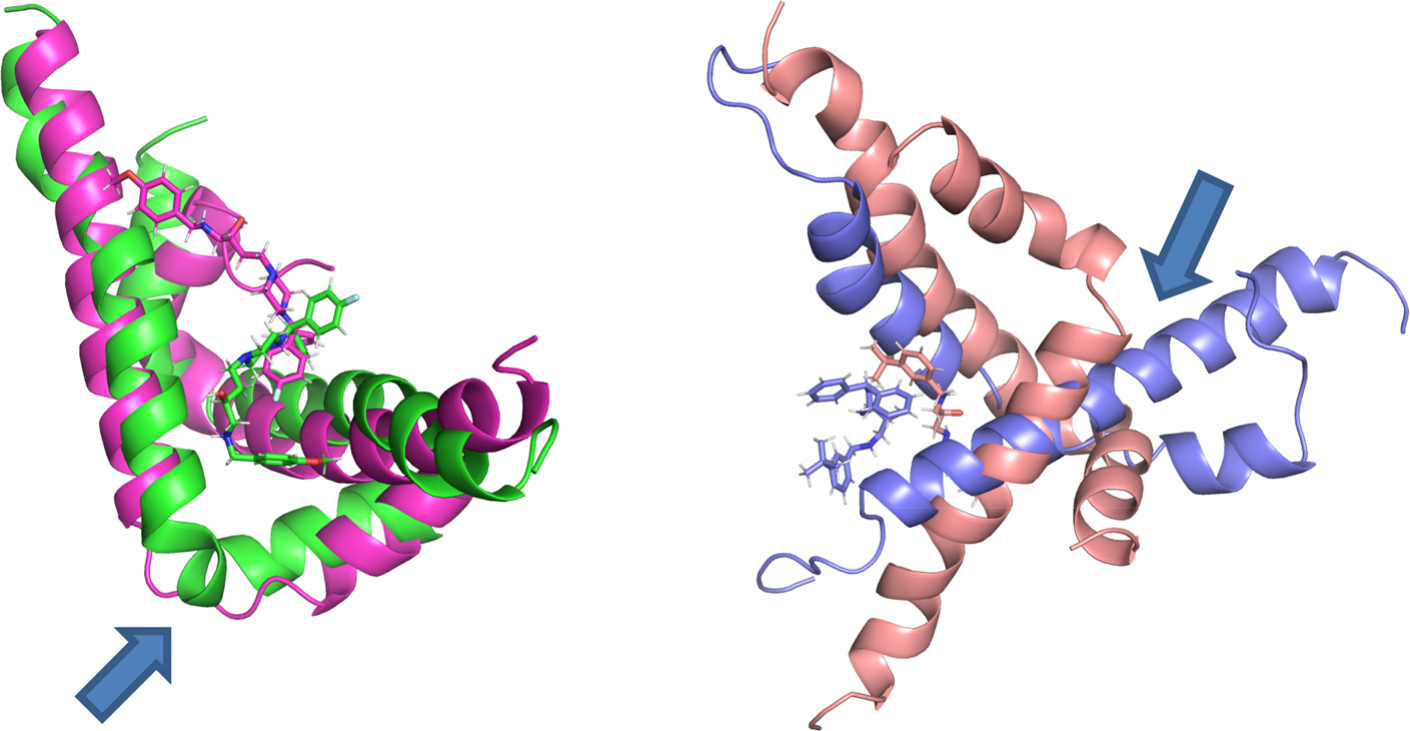
Behavior of the amyloid structure during MD simulation for the system with inactive compound (*S*)-**14** (left) and active compound (*S*)-**18** (right). Blue arrows indicate the most important changes: unraveling of helical chain A for the complex with derivative (*S*)-**14** and reorganization of the helix-kink-helix fragment into a long helix for aggregation inhibitor (*S*)-**18**.

Interactions of compound **18** with K16 and K28 are important, as they could prevent the formation of salt bridges with E22 and D23, which are crucial for the stabilization of amyloid-β fibers. Molecular dynamics simulations showed that, in the case of the ligand-amyloid complex for active compound **18**, chain B underwent reorganization of the helix-kink-helix fragment into a long helix, while for compound **14**, the helical chain A was unraveled (Figure 6).

**Figure 7:**
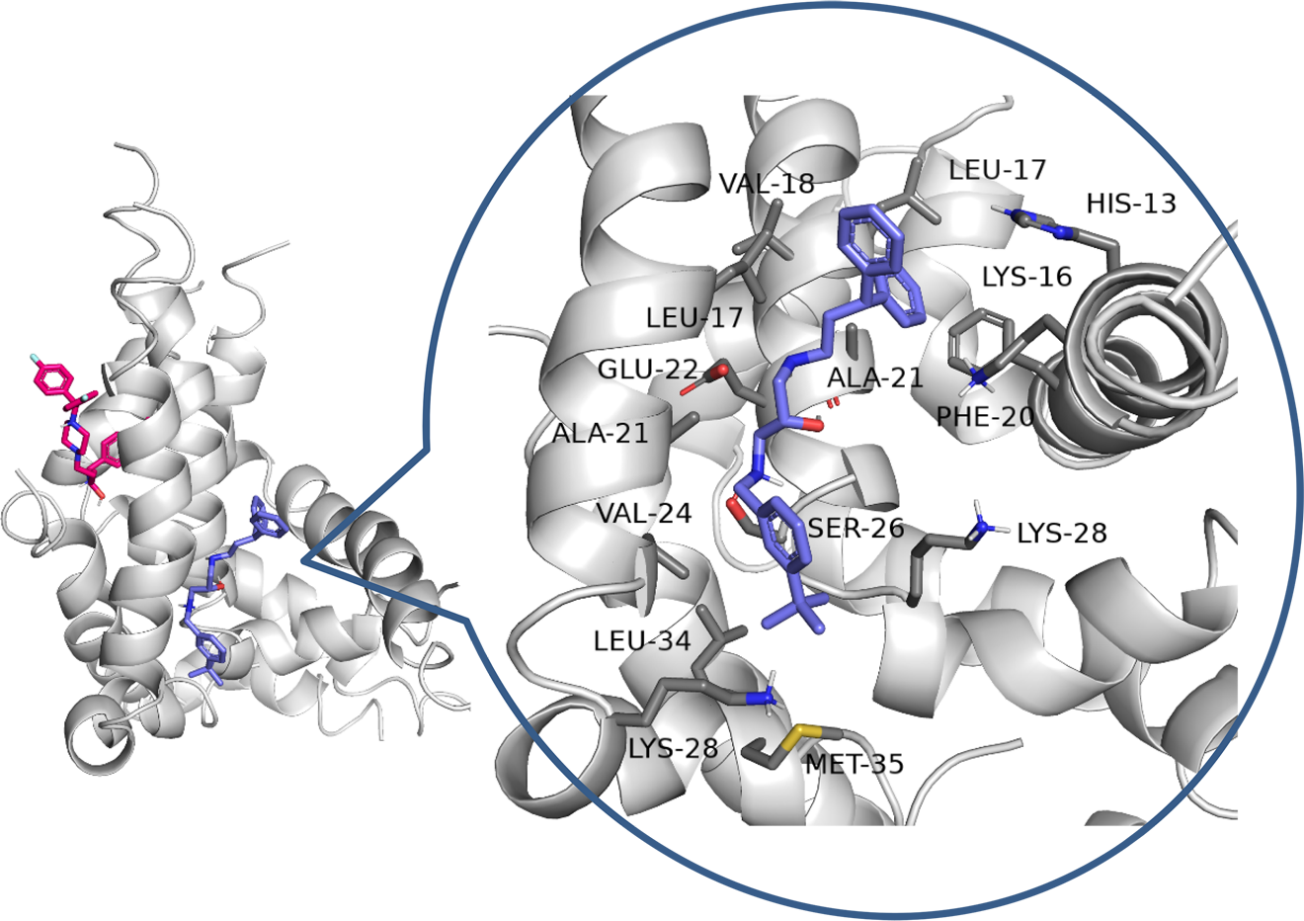
General view of the binding of compounds (*S*)-**18** (violet) and (*S*)-**14** (pink) within the helical pentamer (left). Detailed binding mode of the active derivative (*S*)-**18** (right).

In the case of helical pentamers, the active compounds were bound to a different region compared to that of the inactive compounds (Figure 7). Aggregation inhibitor **18** was located in a cavity formed by chains A, B, and E. This compound formed hydrophobic interactions, especially with L17 and V18 (chain A); H13 and F20 (chain B); and L17, V24, L34, and M35 (chain E). Moreover, hydrogen bond between the hydroxyl group of the inhibitor and the main chain of A21 (chain A), hydrogen bond between the protonated amine and hydroxyl group of S26 (chain A), and ionic interactions between the same amine group and E22 (chain A) were observed. Compound **14** was arranged in the cavity created by chains A, C, D, and E and interacted with surrounding residues. However, this cavity was built of more polar amino acids, and the strength of binding was weaker.

**Figure 8:**
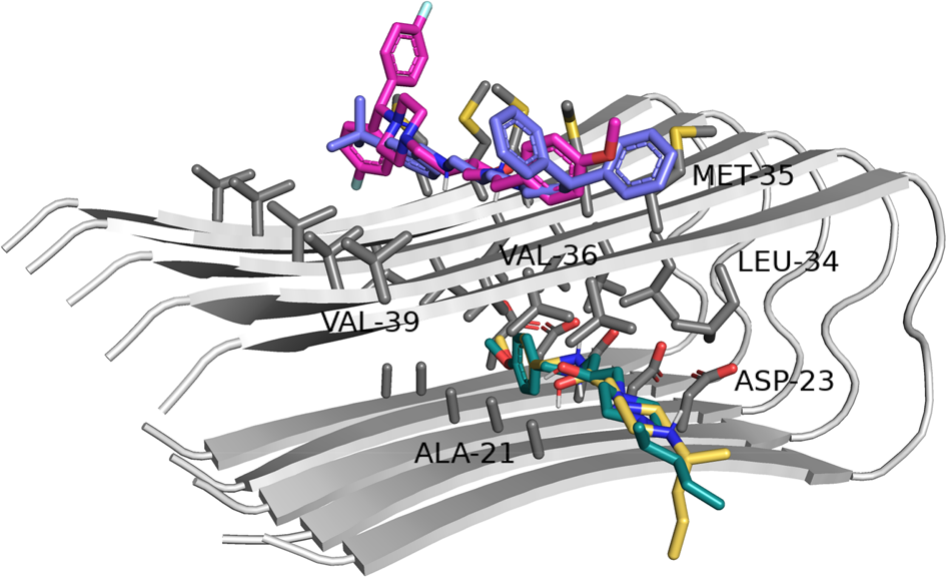
Binding mode of selected compounds with the amyloid-β pentamer of the β-structure (violet = (*S*)-**18**, pink = (*S*)-**14**, yellow = (*S,R*)-**8**, teal blue = (*S,R*)-**1**.

For comparison, the interactions of the tested compounds with the β-sheet form of amyloid-β were evaluated (Figure 8). The docking results showed two possible binding modes, as previously reported for the other compounds from our library.^40^ More branched molecules (**18**, **14**) were docked at the outer side of amyloid fibers, where they were engaged in hydrophobic interactions, mainly with M35 and V39. Fewer branched molecules (usually without diphenylalkyl substituents, e.g., **1**, **8**, and **9**) were located in the lipophilic pocket formed by the side chains of A21, L34, and V36 as well as D23 from each polypeptide chain, where hydrophobic and ionic interactions stabilized their position. Moreover, the protonated amine groups of the ligands interacted with the carboxyl groups of aspartic acid residues.

Even though both active and inactive compounds could bind to amyloid-β in a similar way (both outside and inside the fibers), there was a clear difference in the scoring function value depending on the activity. The more active compounds received higher GoldScore values.

#### 2.2.2 Influence on Tau Protein Aggregation

**Figure 9:**
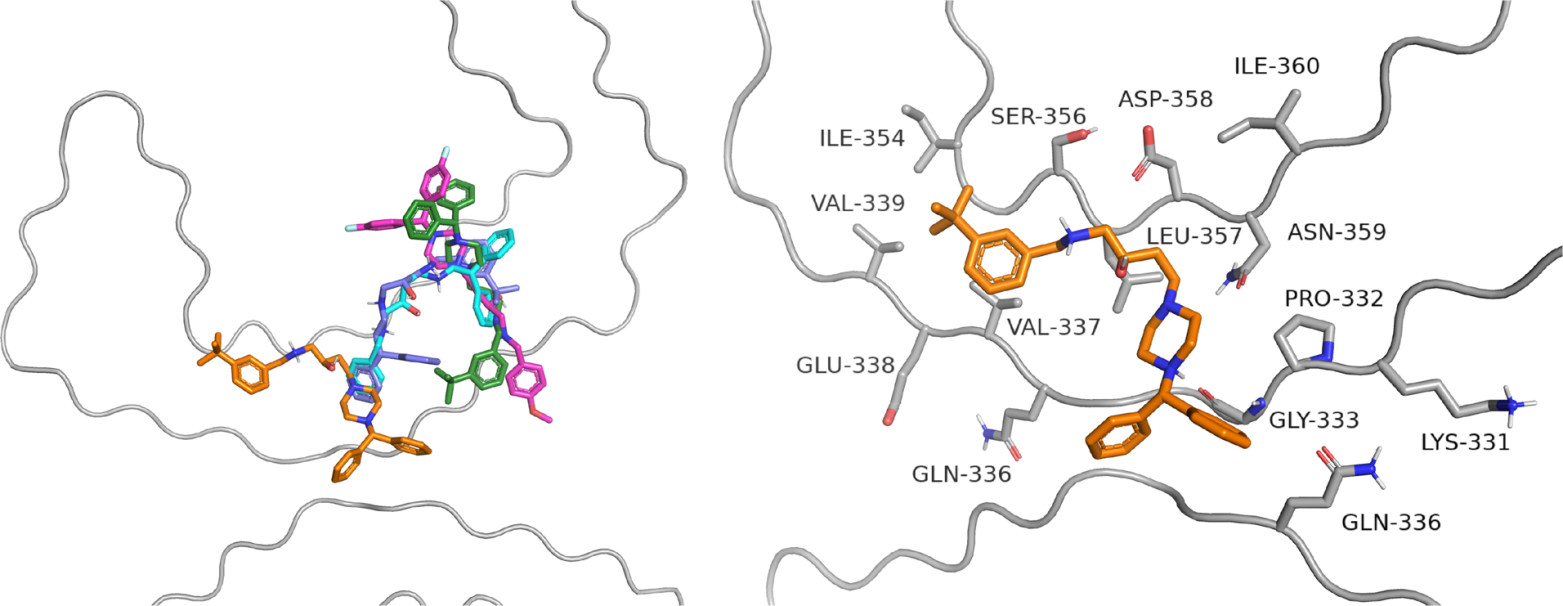
General view of the binding of selected compounds to doublet protofilaments (left panel: green = (*R*)-**6**, orange = (*R*)-**13**, pink = (*R*)-**14**, violet = (*R*)-**18**, blue = (*R*)-**19**). Detailed binding mode of the most active compound (*R*)-**13** (right panel).

To analyze possible interactions between the tested compounds and tau protein, the structure of the doublet protofilament (PDB code: 6VHL), determined by cryoelectron microscopy, was used.^41^ This structure represents the last two residues of repeat R2 (304–305), whole repeats R3 (306–336) and R4 (337–268), and residues K369–K380 in each chain. The docking results revealed that all the tested derivatives could bind to a central part of misfolded tau protein, which may prevent the elongation of filaments (Figure 9). It was observed that the binding of more active inhibitors of the tau aggregation process was stronger as they received higher GoldScore values (e.g., the score values for (*R*)-**6**, (*R*)-**13**, and (*R*)-**14** with inhibition greater than 70% were higher than those for (*R*)-**18** or (*R*)-**19**). Moreover, the *R*-isomers were better assessed than the *S*-isomers, which may demonstrate the higher potency of *R*-isomers against tau aggregation. The most active compound **13** (*R*-isomer) formed hydrophobic interactions via a 3-*tert*-butylbenzyl moiety with the side chains of I354 and V339. The protonated amine group interacted with D358 by ionic bonds. The linker in the case of the *R*-isomer could form hydrophobic interactions with L357. The protonated piperazine ring created a hydrogen bond with the carbonyl group from the main chain of G333. The benzydryl moiety interacted with Q336 residues from both chains.

### 2.3 *In Vitro* Aβ_40_ Kinetic and Aggregation/Dissagregation Studies of Compounds **18** and **21**

**Figure 10:**
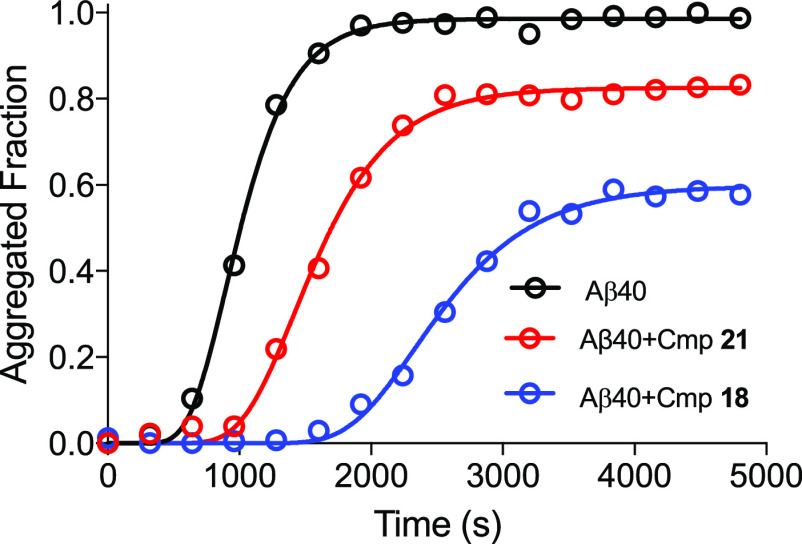
Aβ_40_ amyloid aggregation time-course kinetics tracked by ThT staining. Black = without compound, blue = with compound **18**, and red = with compound **21**. ThT relative fluorescence measurements were performed in triplicate, and the standard errors were less than 5%. The assays were carried out in equimolar concentrations of Aβ_40_ and compounds.

Because amyloid inhibitors may act at several steps of the amyloid aggregation process, we performed kinetic studies using Thioflavin T (ThT) fluorescence assay to determine at which step our compounds inhibit this process (Table 2 and Figure 10). From the most active derivatives, we selected two compounds, **18** and **21**, for these studies. First, we determined their effect on Aβ_40_ aggregation by examining the final fibril amount after 24 h incubation with soluble Aβ_40_ at equimolar concentrations of Aβ_40_ and compounds (10 μM). Then, we performed kinetic studies using the same concentrations. As shown in Table 2, compounds **18** and **21** inhibited Aβ_40_ aggregation at 41.0 and 16.7%, respectively. We also observed a significant decrease in nucleation (*k*_n_) and elongation (*k*_e_) constants for both compounds (Table 2). The 20- and 2-fold decreases in the nucleation ratio for **18** and **21**, respectively, indicate that these compounds directly interact with soluble Aβ_40_ (and potentially with oligomers and prefibrillar species) at the first steps of polymerization. On the other hand, the 2-fold decrease in the elongation ratio for both compounds suggests inhibition of the fibril elongation by interactions with nascent fibers (or in the first β-sheet events). Based on these results, we tried to explain the inhibition differences between these two compounds. We noticed the great difference in the *k*_n_ values for both compounds, while the *k*_*e*_ values were very similar. In summary, it suggests that, while the interaction with preformed fibrils is similar for both compounds, the key difference is in the interaction with soluble Aβ_40_ monomers. Compound **18** displayed a 10-fold ability to interact with Aβ_40_ monomers compared to the ability of **21**, and it could explain the large differences in the thermodynamic final inhibition. Kinetic data is also reflected in the lag (*t*_0_), half (*t*_1/2_), and end (*t*_1_) times of amyloid polymerization. *t*_0_ is increased by ∼1300 and 500 s for **18** and **21**, respectively, as a consequence of the drastic differences in *k*_n_ values. In contrast, increments in *t*_1/2_ and *t*_1_ in comparison to *t*_0_ are similar for both compounds, as a consequence of enclosed *k*_e_ (Table 2). It should be noted that kinetic results are in concordance with *in silico* studies, denoting that these compounds interact with Aβ40 soluble monomers and Aβ40 in a fibrillar conformation.

**Table 2:** Kinetic and Thermodynamic Parameters of Aβ_40_ Amyloid Aggregation and Disaggregation

kinetic parameters	without inhibitor	compound (Cmp.) **18**	Cmp. **21**
*k*_n_ (s^–1^)	3.09 × 10^–5^	1.45 × 10^–6^	1.41 × 10^–5^
*k*_e_ (M^–1^ s^–1^)	486.9	299.5	338.8
*t*_0_ (s)	535	1855	995
*t*_1/2_ (s)	989	2570	1710
*t*_1_ (s)	1444	3284	2424
inhibition%^a^	0.0	41.0	16.7
disaggregation%^b^	0.0	34.3	26.8
disaggregation%^c^	0.0	63.2	56.2

a Kinetic inhibition: [Cmp.] = 10 μM; [Aβ_40_] = 10 μM (1:1).

b Fiber disaggregation: [Cmp.] = 10 μM; [Aβ_40_] = 10 μM (1:1).

c Fiber disaggregation: [Cmp.] = 100 μM; [Aβ_40_] = 10 μM (10:1).

**Figure 11:**
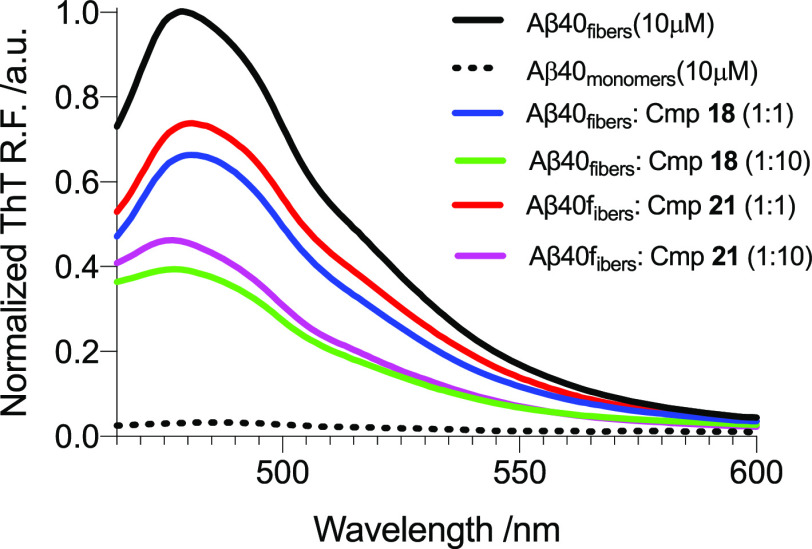
Disaggregation assays. ThT fluorescence of preformed Aβ_40_ fibers after 24 h without or with **18** and **21** at 1:1 (equimolar) and 1:10 (Aβ_40_/Cmp.) ratios. Black–aggregation of Aβ_40_ (positive control); blue = Aβ_40_ with **18** (1:1); green = Aβ_40_ with **18** (1:10); red = Aβ_40_ with **21** (1:1); magenta = Aβ_40_ with **21** (1:10); dashed black = Aβ_40_ monomers (negative control). ThT relative fluorescence measurements were performed in triplicate, and the standard errors were less than 5%.

Finally, we decided to check if our compounds are able to cause disaggregation of preformed fibers. With this goal, we prepared Aβ_40_ fibers in the absence of compounds; then, these fibers were mixed with **18** and **21** at 1:1 and 1:10 ratios (Aβ_40_/compound) and incubated quiescence for 24 h at 4 °C. As shown in Table 2 and Figure 11, both compounds displayed a clear amyloid disaggregation capacity, with **18** being more potent. Site-directed Aβ antibodies, chemical agents such as 4-(2-hydroxyethyl)-1-piperazinepropanesulfonic acid and different chelating molecules, showed disaggregating properties; nevertheless, their disaggregating mechanisms have not yet been completely clarified.^42−45^ Recently it was reported, for molecular tweezer CLR01, with a disaggregation ability against amyloid proteins such as the prion protein (PrP), that the direct binding to lysine residues could interrupt oligomerization.^46^ Further studies with CLR01, using a long time scale of a molecular dynamic simulation, revealed the potential mechanism of disaggregation of PrP. It demonstrated the binding of CLR01 with K222 nitrogen by the π–cation interaction of its aromatic rings and the formation of a salt bridge/hydrogen bond of one of the two rotatable peripheral anionic phosphate groups.^47^ As reported in *in silico* studies, inhibitor **18** was able to create a wide range of interactions with amyloid-β. Ligand–peptide interactions, such as cation−π with K28 (chain A) and F20 (chain B), as well as a hydrogen bond with K16, could compete with interactions between amyloid chains. This could be a potential explanation of its disaggregation ability.

## 3 Conclusions

The lack of an effective AD treatment creates a necessity for new clinical candidates, preferably addressing processes underlying the disease. Misfolding and aggregation of Aβ and tau proteins are undoubtedly such processes. Therefore, in our studies, we focused on the development of new multifunctional ligands that would affect different stages of these processes. Using *in cellulo*, *in silico*, and *in vitro* methods, we did not only reveal unique biological activities but also showed possible compound–protein interactions.

In the present extended *in cellulo* studies directed at Aβ and tau aggregation inhibition, we found very potent dual aggregation inhibitors. To the best of our knowledge, the activity of these compounds is higher than that of multifunctional ligands reported to date.^10^ Among the tested derivatives, we selected compound **18**, which exhibited a unique profile of biological activity. This compound is the most potent and balanced dual aggregation inhibitor (Aβ_42_ inh. 80.0%, tau inh. 68.3% in 10 μM), with previously reported inhibitory activity against *h*BuChE, *h*BACE1, and Aβ *in vitro* (*h*BuChE IC_50_ = 5.74 μM; *h*BACE1 IC_50_ = 41.6 μM; Aβ aggr. inh. IC_50_ = 3.09 μM).^23^

In docking studies, we tried to explain the different structural requirements for the inhibition of Aβ vs tau that we observed from the *in cellulo* results. Docking studies for Aβ showed that our compounds form hydrophobic interactions with amyloid and inhibit the aggregation process by the stabilization of the α-helical structure of amyloid. However, for tau, inhibition was possible, because our compounds bound to a central part of the misfolded tau protein and prevented the elongation of filaments. Moreover, in docking studies, we took into account the enantiomers (the biological assays were performed for racemic mixtures). The data suggest the significant impact of chirality on antiaggregating activity; we predicted that *S*-isomers are favorable for Aβ and *R*-isomers for tau inhibition. All these results may help to design the next generations of dual or selective aggregation inhibitors.

According to the kinetic data, our selected compounds could act on several steps of amyloid aggregation process: They do not only inhibit the aggregation by interacting with soluble Aβ_40_ but also slow down the elongation of new fibers. These observations are in line with results obtained in docking studies. It needs to be highlighted that, apart from the ability to inhibit amyloid aggregation, these compounds displayed disaggregating properties and therefore can fully prevent the formation of amyloid plagues.

In summary, our results from extended *in cellulo*, *in silico*, and *in vitro* studies may serve as a good starting point for further research either on Aβ and tau aggregation processes or on their inhibitors/modulators.

## 4 Experimental Section

### 4.1 Evaluation of the Inhibitory Activity against Aβ_42_/Tau Aggregation in *E. coli* Overexpressing Aβ_42_/Tau

The procedure of the assay was described previously in refs (24) and (31). All chemicals were purchased from Sigma-Aldrich, and reagents for bacterial media were purchased from Pronadisa (Sevilla, Spain). M9 minimal medium for 100 mL: 10 mL of 10× salts (0.68 g of Na_2_HPO_4_, 0.30 g of KH_2_PO_4_, 0.05 g of NaCl, and 0.10 g of NH_4_Cl), 0.2 mL of 1 M MgSO_4_, 0.2 mL of 50 mM CaCl_2_, 2.5 mL of 20% glucose, and 87.1 mL of H_2_O. As the reference compound, we used **DP-128** (*N*-{8-[(6-chloro-1,2,3,4-tetrahydroacridin-9-yl)amino]octyl}-5-(4-chlorophenyl)-1,2,3,4-tetrahydrobenzo[h][1,6]naphthyridine-9-carboxamide).^27,33^

#### 4.1.1 Aβ_42_ Aggregation Inhibition Assay in Bacterial Cells

*E. coli* BL21 (DE3) competent cells were transformed with the pET28a vector (Novagen, Inc., Madison, WI, USA) carrying the DNA sequence of Aβ42. To prepare the overnight culture, 10 mL of M9 minimal medium containing 50 μg/mL kanamycin and 25 μM thioflavin ThS was inoculated with a colony of BL21 (DE3) bearing the plasmid Aβ_42_. To reach an OD_600_ of approximately 2–2.5, the cultures were grown overnight at 37 °C and 180 rpm using an incubator shaker (Ovan, Barcelona, Spain). Then, 200 μL of overnight culture was transferred into 1.5 mL Eppendorf tubes containing 790 μL of fresh M9 minimal medium with 50 μg/mL kanamycin, 25 μM ThS, and 10 μL of reference or tested compounds (1 mM in DMSO, final concentration: 10 μM) or DMSO. For the expression of Aβ_42_, 1 mM isopropyl 1-thio-β-D-galactopyranoside (IPTG) was added to each Eppendorf tube. To determine the minimal level of Aβ_42_, positive controls without IPTG were prepared. The maximum level of Aβ_42_ was evaluated in the negative controls (the induced IPTG samples with DMSO without inhibitor). All resulting cultures were grown for 24 h at 37 °C and 180 rpm using an Ovan incubator shaker. For the inhibitory activity evaluation, 200 μL of each Eppendorf tube was transferred in triplicate into a 96-well plate. The fluorescence emission at 485 nm, when excited at 430 nm, was recorded using a multimode microplate reader (Beckman Coulter). To control the level of bacterial growth and exclude the potential intrinsic toxicity of the tested compounds, the absorbance at 620 nm (OD_620_) of these samples was monitored. Three independent experiments were conducted, and the final data were calculated as their average.

#### 4.1.2 Tau Aggregation Inhibition Assay in Bacterial Cells

*E. coli* BL21 (DE3) competent cells were transformed with pTARA containing the RNA polymerase gene of T7 phage (T7RP) under the control of the promoter pBAD. Then, the resulting cells were transformed with the pRKT42 vector encoding four repeats of tau protein in two inserts. To prepare the overnight culture, 10 mL of M9 minimal medium containing 50 μg/mL ampicillin, 12.5 μg/mL chloramphenicol, and 25 μM ThS was inoculated with a colony of BL21 (DE3) bearing the tau plasmids. To reach an OD_600_ of approximately 2–2.5, the cultures were grown overnight at 37 °C and 180 rpm using an incubator shaker (Ovan, Barcelona, Spain). Then, 200 μL of overnight culture was transferred into 1.5 mL Eppendorf tubes containing 790 μL of fresh M9 minimal medium containing 50 μg/mL ampicillin, 12.5 μg/mL chloramphenicol, 25 μM ThS, and 10 μL of reference or tested compounds (1 mM in DMSO, final concentration: 10 μM) or DMSO. For the expression of tau protein, 0.25% arabinose was added to each Eppendorf tube. To determine the minimal level of tau protein, positive controls without arabinose were prepared. The maximum level of tau protein was evaluated in the negative controls (the samples with DMSO, 0% inhibition). All resulting cultures were grown for 24 h at 37 °C and 180 rpm using an Ovan incubator shaker. For the inhibitory activity evaluation, 200 μL of each Eppendorf tube was transferred in triplicate into a 96-well plate. The fluorescence emission at 485 nm, when excited at 430 nm, was recorded using a multimode microplate reader (Beckman Coulter). To control the level of bacterial growth and exclude the potential intrinsic toxicity of the tested compounds, the absorbance at 620 nm (OD_620_) of these samples was monitored. Three independent experiments were conducted, and the final data were calculated as their average.

### 4.2 *In Silico* Studies

#### 4.2.1 Amyloid Aggregation

##### 4.2.1.1 Ligand Preparation

Compounds in SMILES format were converted into three-dimensional structures using the LigPrep module from Schrodinger Suite. All possible stereoisomers were generated, and the ionization states at pH 7.4 ± 0.2 were predicted with Epik. Upon optimization in a water environment using Macromodel with an OPLS3 force field, the ligand structures were saved in mol2 file format.

##### 4.2.1.2 Amyloid Preparation

Amyloid-β structures (PDB codes: 1IYT, 2BEG) were downloaded from the Protein Data Bank. The first conformation for each structure was selected and further preprocessed with Protein Preparation Wizard from Schrodinger Suite. The structures were further utilized for the analysis of ligand binding with helical monomeric and β-sheet pentameric forms of amyloid-β. Moreover, the helical monomer 1IYT was used to build oligomeric structures using PyDock3 protein–protein docking tools. The final conformations of the helical dimer, trimer, tetramer, and pentamer were selected based on energetic criteria and further processed with Protein Preparation Wizard, including restrained minimization with the OPLS3 force field and convergence of heavy atoms to RMSD 1.0. All amyloid-β forms were saved as pdb files for docking.

##### 4.2.1.3 Docking

Molecular docking was performed with GOLD Suite 5.1. All ligands were docked to helical monomers, dimers, and pentamers, as well as pentamers of β-sheet structures, using standard settings of a genetic algorithm with population sizes of 100, 5 islands, and 100 000 operations. The binding site included the whole protein to test where ligands can bind in a preferential way. For each compound, 10 poses were generated and assessed by the GoldScore function. The results were analyzed with Hermes 1.5 and PyMOL 2.3.4.

##### 4.2.1.4 Molecular Dynamics Simulations

The ligand–protein complexes obtained from docking were used to build the systems for MD simulations with Charmm-Gui. Ligands were parametrized with the Charmm general force field. The size of the rectangular waterbox (TIP3P) was fitted to the protein size with an edge distance of 20 Å. Sodium and chloride ions were added to neutralize the system and reach a physiological environment of 0.15 M NaCl. Periodic boundary conditions and particle mesh Ewald (PME) electrostatics were used. All calculations were performed with NAMD 2.10 and the CHARMM36m force field. The SHAKE algorithm was applied for rigid bonds. The system with restrained protein and ligand was equilibrated, including minimization (10 000 steps) and the subsequent 250 ps NVT dynamics simulation with a 2.0 fs time step at 303.15 K. Finally, a 10 ns MD run was performed applying the NPT ensemble with a 2.0 fs time step, pressure 1013.25 hPa, and temperature 303.15 K. Langevin dynamics were used to control the temperature. The trajectories from MD simulations were analyzed using VMD 1.9.2.

#### 4.2.2 Tau Protein Aggregation

##### 4.2.2.1 Tau Protein Preparation

The structure of paired helical filaments from Alzheimer’s disease human brain tissue was obtained from the Protein Data Bank (PDB code: 6VHL). The fragment containing 304–380 amino acid residues was prepared for docking with Hermes 1.5 in a default manner.

##### 4.2.2.2 Docking

All ligands used to study interactions with amyloid-β were also docked to fragments of tau protein. The settings of docking and the analysis were conducted in an analogous way to that regarding amyloid-β.

### 4.3 *In Vitro* Studies

#### 4.3.1 Preparation of Aggregate-Free Amyloid-β Peptide

Aβ_40_ was purchased from Bachem (Switzerland). For the preparation of aggregate-free amyloid-β peptide, Aβ_40_ (1 mg) was solubilized in 1,1,1,3,3,3-hexafluoro-2-propanol (HFIP; 500 μL) under vigorous stirring at room temperature for 1 h. The resulting solution was sonicated for 30 min and subsequently stirred at room temperature for an additional hour. The solution was then maintained at 4 °C for 30 min to avoid solvent evaporation during aliquot collection. To eliminate possible insoluble materials, the samples were filtered over 0.22 μm filters. Finally, aliquots of soluble Aβ_40_ were collected, and HFIP was evaporated under a gentle N_2_ stream. The samples were stored at −33 °C.

#### 4.3.2 Aβ_40_ Aggregation Assays

The samples were resuspended in 50 μL of DMSO, and the monomers were solubilized through sonication for 10 min. Native Buffer (940 μL; 50 mM Tris·HCl at pH 7.4 and 150 mM NaCl) was added, and the samples were divided in four parts (247.5 μL). Finally, 2.5 μL of each compound at 1 mM in DMSO (obtaining a final concentration of 10 μM) or DMSO without compound (positive control) was added. The final concentration of Aβ_40_ was 10 μM. The samples were placed in a thermomixer (Eppendorf, Germany) at 37 °C and stirred at 1400 rpm. At 24 h, 135 μL of sample were mixed with 15 μL of ThT at 250 μM, obtaining a final ThT concentration of 25 μM. Finally, the aggregation was tracked by detecting ThT fluorescence (λ_exc_ = 445 nm; λ_em_ = 480 nm) using an Aminco Bowman Series 2 luminescence spectrophotometer (Aminco-Bowman AB2, SLM Aminco, Rochester, NY, USA).

#### 4.3.3 Aβ_40_ Kinetic Aggregation Assays

Aβ_40_ was suspended in 50 μL of DMSO using a sonication bath for 10 min. To evaluate the effect of compounds on peptide aggregation, the volume was completed adding 950 μL of Native Buffer (50 mM Tris·HCl, 150 mM NaCl, pH 7.4) containing ThT and each compound at final concentrations of 25 and 10 μM, respectively. The final concentration of Aβ_40_ was 10 μM. Each sample (200 μL) was placed in a 96-wall grenier standard platelet and incubated under strong agitation at 37 °C. The ThT fluorescent signal was checked each 5 min using a ClarioStar Plus plate reader (BMG Labtech, Germany) at an excitation wavelength of 445 nm and an emission wavelength of 480 nm.

#### 4.3.4 Aβ_40_ Kinetic Aggregation Analysis

The amyloid Aβ_40_ aggregation may be analyzed as an autocatalytic reaction using eq 1
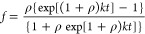
1where *f* is the fraction of Aβ peptide in the fibrillar/aggregated form, the rate constant *k* includes the kinetic contributions arising from the formation of the nucleus from monomeric Aβ and the elongation of the fibril, which are described by rate constants *k*_n_ and *k*_e_, respectively, and ρ is a dimensionless parameter that describes the ratio of *k*_n_ to *k*. Equation 1 is obtained under the boundary conditions of *t* = 0 and *f* = 0, where *k* = *k*_e_*a* (*a* is the protein concentration). By nonlinear regression of *f* against *t*, values of ρ and *k* were obtained, and from them, the rate constants *k*_e_ (elongation constant) and *k*_n_ (nucleation constant) were obtained. The extrapolation of the linear portion of the sigmoid curve to abscissa (*f* = 0), and to the highest ordinate value of the fitted plot, afforded two values of time (*t*_0_ and *t*_1_), which corresponded to the lag time and to the end-time reaction.^27,48^ The time at which half of the protein was aggregated (i.e., when *f* = 0.5) was considered the time of half aggregation (*t*_1/2_). The analysis was performed using the GraphPad Prism 8.3.0 for OS X.

#### 4.3.5 Aβ_40_ Fibril Disaggregation Assays

The samples were resuspended in 50 μL of DMSO, and the monomers were solubilized through sonication for 10 min. Native Buffer (940 μL; 50 mM of Tris·HCl at pH 7.4 and 150 mM of NaCl) was added, and the samples were divided into four parts (247.5 μL). The samples were placed in a thermomixer (Eppendorf, Germany) at 37 °C and stirred at 1400 rpm. After fibril formation, 2.5 μL of each compound at 1 mM and 10 mM in DMSO (obtaining final concentrations of 10 and 100 μM, respectively) or DMSO without compound (positive control) was added. The final concentration of Aβ_40_ was 10 μM. The samples were stored in quiescence for 24 h at 4 °C. At 24 h, 135 μL of sample was mixed with 15 μL of ThT at 250 μM, obtaining a final ThT concentration of 25 μM. Finally, the aggregation was tracked by detecting ThT fluorescence (λ_exc_ = 445 nm; λ_em_ = 480 nm) using an Aminco Bowman Series 2 luminescence spectrophotometer (Aminco-Bowman AB2, SLM Aminco, Rochester, NY, USA).
